# The Chinese version of story recall: a useful screening tool for mild cognitive impairment and Alzheimer’s disease in the elderly

**DOI:** 10.1186/1471-244X-14-71

**Published:** 2014-03-10

**Authors:** Jing Shi, Mingqing Wei, Jinzhou Tian, Julie Snowden, Xuekai Zhang, Jingnian Ni, Ting Li, Wenjia Jian, Congcong Ma, Yanping Tong, Jianping Liu, Tonghua Liu, Pengwen Wang, Yongyan Wang

**Affiliations:** 1 The 3rd Department of Neurology, Dongzhimen Hospital, Beijing University of Chinese Medicine, Beijing 100700, China; 2 Cerebral Function Unit, Greater Manchester Neuroscience Centre Salford Royal NHS Foundation Trust, Salford M6 8HD, UK; 3 Center for Evidence-Based Chinese Medicine, Beijing University of Chinese Medicine, Beijing 100029, China; 4 Beijing University of Chinese Medicine, Beijing 100029, China; 5 Key Laboratory of Chinese Internal Medicine, Ministry of Education, Beijing University of Chinese Medicine, Beijing, China; 6 Institute of Clinical Medicine, China Academy of Chinese Medical Sciences, Beijing, China

**Keywords:** Alzheimer’s disease, Mild cognitive impairment, Delayed story recall, Sensitivity, Specificity

## Abstract

**Background:**

Decline in verbal episodic memory is a core feature of amnestic mild cognitive impairment (aMCI). The delayed story recall (DSR) test from the Adult Memory and Information Processing Battery (AMIPB) discriminates MCI from normal aging and predicts its conversion to Alzheimer’s dementia. However, there is no study that validates the Chinese version of the DSR and reports cut-off scores in the Chinese population.

**Methods:**

A total of 631 subjects were screened in the memory clinics of Dongzhimen Hospital, Beijing University of Chinese Medicine, China. 249 were considered to have normal cognition (NC), 134 met diagnostic criteria for MCI according to the MCI Working Group of the European Consortium on Alzheimer's Disease, and 97 met criteria for probable Alzheimer’s disease (AD) according to the NINCDS/ADRDA criteria, 14 exhibited vascular dementia (VaD), and 50 had a diagnosis of another type of dementia. Receiver operating characteristic (ROC) curve analyses were used to calculate the story recall cutoff score for detecting MCI and AD. Normative data in the NC group were obtained as a function of age and education.

**Results:**

In this Chinese sample, the normative mean DSR score was 28.10 ± 8.54 in the 50–64 year-old group, 26.22 ± 8.38 in the 65–74 year-old group, and 24.42 ± 8.38 in the 75–85 year-old group. DSR performance was influenced by age and education. The DSR test had high sensitivity (0.899) and specificity (0.799) in the detection of MCI from NC using a cut-off score of 15.5. When the cutoff score was 10.5, the DSR test obtained optimal sensitivity (0.980) and specificity (0.938) in the discrimination of AD from NC. Cutoff scores and diagnostic values were calculated stratified by age and education.

**Conclusions:**

The Chinese version of the DSR can be used as a screening tool to detect MCI and AD with high sensitivity and specificity, and it could be used to identify people at high risk of cognitive impairment.

## Background

Mild cognitive impairment (MCI), a diagnosis given to individuals who have memory or slight cognitive impairments but do not meet the criteria for dementia, is a transitional stage between normal aging and dementia [1]. The prevalence of MCI ranges from 16% to around 31% in elderly people (older than 65 years), with approximately 15% of people with MCI converting to Alzheimer’s disease (AD) within one year, 34% within two years, and 57% in 3 years [2]. Among different types of MCI, amnestic MCI is recognized to progress preferentially to AD, and is recognized as a possible prodromal stage of AD [3]. Studies have shown that 5.9% of people aged 65 years and above in China have AD, at present China has 6 million patients with dementia and, with a rapidly ageing population [4], it is estimated to have 1 million new cases every year [5]. However, an international team of researchers has found that over 93.1% of dementia cases in China go undetected, with a high level of undiagnosed dementia in rural areas [6], which is mainly due to lack of neuropsychological assessment instruments applicable to the Chinese language and culture.

In the detection of MCI, verbal episodic memory performance is generally considered as the best predictor of cognitive decline [7]. Previous studies have shown that episodic memory tests such as paragraph recall are sensitive to MCI and very early cognitive impairment in older adults [8]. Delayed story recall performance can not only significantly predict progression from MCI to AD [9,10], but also has high sensitivity and specificity for the early diagnosis of AD, and can discriminate very mild/early stage AD and non-demented elderly effectively [11]. Delayed memory testing and recall-based assessments have been identified as the most discriminating factors in those individuals at risk of progression to AD [12]. To date, there is no consensus on the optimal neuropsychological assessment tool to assess episodic memory in MCI. The Adult Memory and Information Processing Battery [AMIPB] [13] is a tool that was designed to assess immediate registration of verbal information and retention over time. It contains six sub-tests: two verbal memory tests (one of which is a story recall), two visual memory tests and two information-processing tests. The story recall test includes immediate story recall (ISR) and delayed story recall (DSR), and is similar in structure to the Wechsler Memory Scale (WMS) logical memory test [14]. Previous studies have shown that the story recall test is the strongest predictor of reported memory performance in daily life by elderly adults [15,16]. The advantages of the AMIPB story recall test is that it has detailed administration and scoring instructions, and normative data ranging from 18 to 75 years old.

However, most studies have been conducted in the setting of a Western culture. There is no Chinese version nor any studies using the story recall test in China. Moreover, cut-off scores for the Chinese population are not known. Hence, it is important to validate the subtests in the context of the Chinese culture and language, and develop appropriate cut-off points for the Chinese population.

This study aims to evaluate the sensitivity and specificity of the Chinese version of the delayed story recall test, and to determine the optimal cutoff score for a clinical diagnosis of amnestic MCI or AD in the Chinese population.

## Methods

### The Chinese version of the story recall

The story from the AMIPB, which is about a woman and a thief, was translated into Chinese using back translation methods. The original English version was translated by clinical psychologists, one bilingual, and all with an excellent command of English; the translations were then handed to two specialists in neuropsychology, and two psychiatrists for their revision; they discussed and revised the first version translations; back translations were then made from Chinese into English by a bilingual psychologist; finally, a final Chinese version was made by clinical neuropsychology and psychiatry professionals.

The Chinese version represents a literal translation of the original English version, with the exception of the following modifications: We replaced the name ‘Angela/Harper’ with the Chinese name ’Shuzhen Wang (王淑珍)’.

### Participants

Chinese-speaking adults, aged 50 to 85 years old, with memory complaints, were screened between January 2007 and April 2011 in the memory clinic of Dongzhimen Hospital of Beijing University of Chinese Medicine, China.

All participants underwent a clinical and neuropsychological evaluation, involving the following assessment instruments: the Mini-mental state examination (MMSE) [17], Activities of Daily Living (ADL) [18] scale, the Hachinski Ischemia scale (HIS) [19], the Hamilton Depression Scale (HAMD) [20], the Adult Memory and Information Processing Battery (AMIPB) story recall [13], and the Clinical Dementia Rating (CDR) [21] score. The allocation of patients to different groups was based on results of the mental state examination, neuropsychological assessment, laboratory results and neuroimaging.

Normal control (NC) subjects were identified in accordance with criteria used in the Mayo research study [22]: (1) no active neurological or psychiatric disease, (2) no psychotropic medication, (3) no medical disorder for which the disorder or its treatment could compromise cognitive function, an MMSE score >26 point (for people attaining higher education), MMSE>23 (middle school), MMSE>22(primary school), MMSE>19 (Illiteracy), scores based on a previous study of the Chinese population [17,23], CDR [21] score = 0;(4) fully independent abilities in activities of daily living (ADL<16) [18].

MCI subjects were required to meet diagnostic criteria for MCI documented by the MCI Working Group of the European Consortium on Alzheimer's Disease [24]. The following were adopted as operational criteria (Chinese version) for inclusion into the MCI group for the present study at screening [23]: (1) cognitive complaints from the patients or their families; (2) report of a relative decline in cognitive functioning during the past year by the patient or informant; (3) normal general cognitive function, as determined by a clinician's judgment based on a structured interview with the patients: MMSE scores in the normal control ranges; (4) cognitive disorders as evidenced by clinical evaluation, CDR [21] score = 0.5, memory domain = 0.5; (5) preservation of activities of daily living, ADL score <16 [18]; (6) absence of dementia, not sufficiently impaired, cognitively and functionally, to meet NINCDS-ADRDA criteria for AD [25], as judged by an experienced dementia research clinician. (7) In addition, they were judged to have a score of ≤12/17 on the HAMD scale [20], of ≤4 on the HIS [19], and no or minimal medial temporal atrophy (MTA) or hippocampal volume atrophy on an MRI scan. The latter was assessed using the medial temporal lobe atrophy (MTA) scale, the most widely published visual rating scale. Ratings were made by three clinicians who were blinded to diagnosis and age of the subjects, and a definitive score was assigned through consensus [23].

Exclusion criteria were: (1) meeting criteria for dementia; (2) depression or psychosis of juvenile onset; (3) other neural system diseases including Parkinson's disease, or other cerebral pathology as verified by a formal clinical examination.

The diagnosis of dementia was based on the Diagnostic and Statistical Manual of Mental disorders, fourth edition (DSM-IV) [26], and the diagnosis of Alzheimer’s disease was in accordance with the National Institute of Neurological Communicative Disease and Stroke (NINCDS) and Alzheimer's Disease and Related Disorders Association (ADRDA) criteria for probable AD [25]. A diagnosis of AD was based on clinical and neuropsychological assessments:, MMSE scores fall below the normal, education-appropriate cutoffs; (2) two or multiple domain cognitive impairment, CDR ≥ 0.5; (3) progressive deterioration of memory and other cognitive functions; (4) no disturbance of consciousness; (5) impairment in activities of daily living, ADL score ≥16; (6) absence of cerebrovascular disease, HIS score ≤4; (7) and medial MTA or hippocampal atrophy on MR imaging; (8) exclusion of other disease which may cause cognitive impairment.

AD exclusion criteria were: (1) acute onset; (2) focal nervous system signs in early stage disease, for example, incomplete paralysis, anesthesia, visual field defect, and ataxia; (3) epileptic attack or gait disturbance in early stage of disease; (4) depression or other mental disorders, HAMD>12 (17 items).

**Figure 1:**
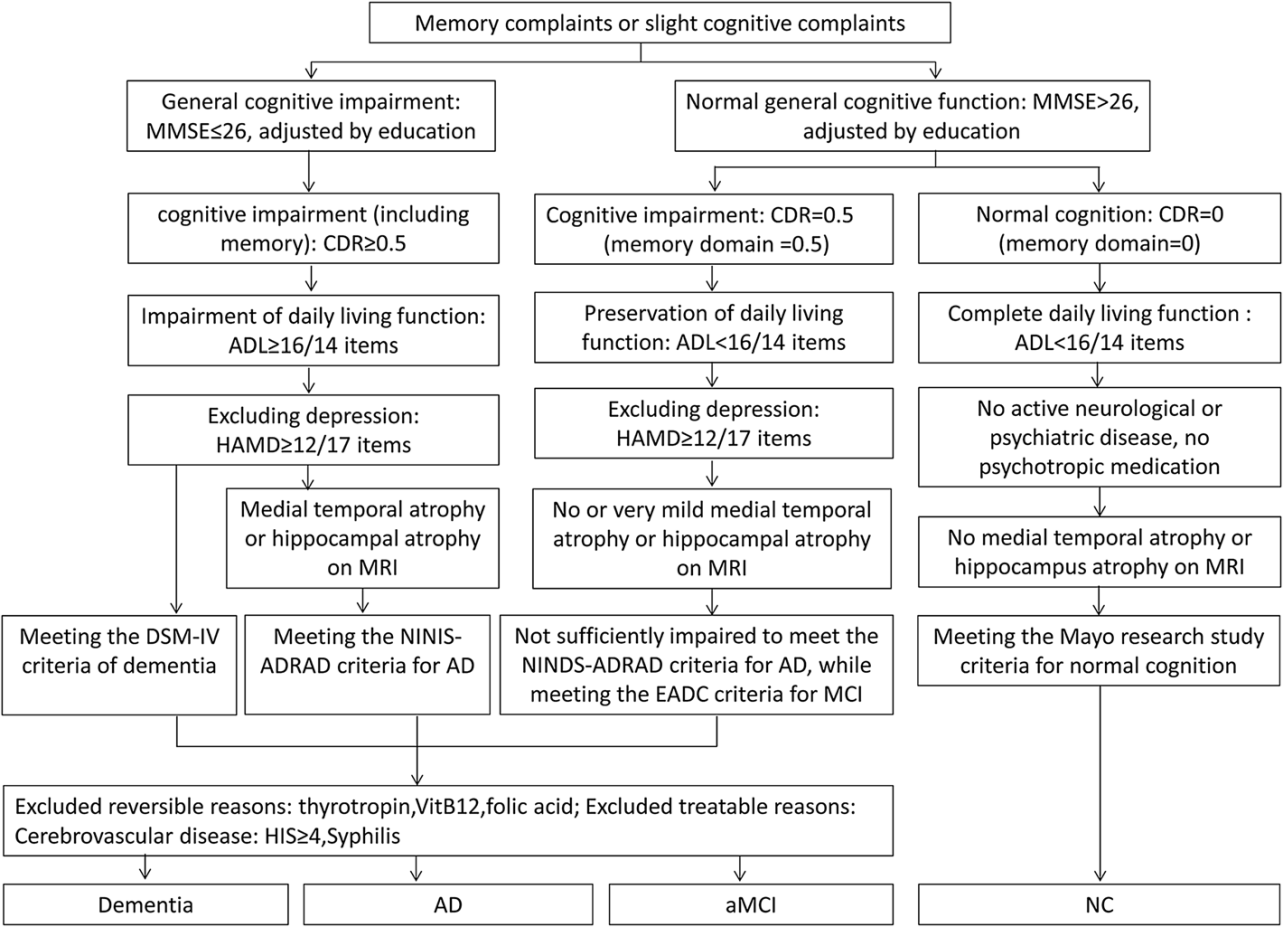
**Diagnostic algorithm for Alzheimer’s disease, amnestic MCI, and normal cognition.** Notes: AD = Alzheimer’s disease; aMCI = amnestic mild cognitive impairment; CDR = clinical dementia rating scale; MMSE = Mini-mental State Examination; NC = normal cognition; ADL = Activities of Daily Living; HIS = Hachinski Ischemia scale; HAMD = Hamilton Depression Scale.

The operationalized criteria for inclusion of amnestic MCI and AD have been reported previously [27]. The diagnostic flow chart is shown in Figure 1.

### Procedures

The clinical assessments, laboratory investigations, measurements of vital signs (including temperature, blood pressure and electrocardiogram), neurological tests (including an examination of cranial nerves, motor coordination, muscle power and tone) and neuroimaging were carried out before the patients were enrolled in the study. Before initiation of the project, 6 physicians participated in training in standard administration of the DSR. The participating physicians were instructed to administer the DSR consistent with the original guidelines of the AMIPB, outlined below.

A short story is read to the patient, who is then asked to immediately recall it. Patients are allowed up to 2 minutes to recall the story and are allocated a score of 0, 1 or 2 depending on the accuracy of their recall. Under the AMIPB scoring scheme, any correctly recalled idea (or an accurate paraphrase thereof) is awarded two points, and any vaguely or partially recalled ideas receive one point. Detailed guidelines with examples of scoring are also given in the AMIPB. The story contains 28 ideas. Therefore the maximum possible score is 56. After 23–30 minutes the patient is asked to recall the same story; the same scoring system is applied.

The psychiatric and neurological classifications were made blind to subjects’ story recall performance. The story recall test score had no impact on diagnostic classifications of AD, MCI or NC.

The protocol was approved by Dongzhimen Hospital, Beijing University of Chinese Medicine Institutional Ethics Committee. The study was undertaken in accordance with the principles of the Declaration of Helsinki. The patients and responsible caregivers provided written informed consent.

### Statistical methods

SPSS 17.0 for Windows was used for the data analyses. Sex distributions in the 3 groups were compared using the chi-square test, mean age, education years, MMSE scores and DSR were compared by nonparametric tests. Partial correlations were determined between the MMSE and DSR by controlling for age and education. Receiver operating characteristic (ROC) curve analyses allowed calculation of the optimal sensitivity (to correctly detect cases) and optimal specificity (to correctly detect controls) using different cut-off scores of the DSR. The positive predictive values (ppv) and negative predicative (NPV) values were measures at the threshold scores. A proportion of the study cohort was re-assessed 3 months after the initial test, and bivariate correlation analysis was applied to evaluate retest reliability of the DSR. P values below 0.05 were considered statistically significant throughout the analysis.

A previous study has shown that DSR performance was influenced by age and education. Accordingly, we attempted to develop a statistical correction for effects of age and education and test the efficacy of the statistically adjusted DSR as a screening test for detecting NC, MCI, AD and dementia.

The following steps were taken to convert raw scores to T scores: (1) multiple regression based norms were constructed using the normal cognition group; (2) using the weights (beta’s) from the same regression analysis, expected scores for each patient were calculated using the formula (expected value = 31.535 + 0.746 × years of education-0.206 × age); (3) the residual of each case was calculated using the raw score minus expected score; (4) the standardized residuals (Z values) was calculated using the residuals/residual Std.Deviation; (5) the standardized residuals (Z values) was converted to T scores according to the formula: T scores = standardized residual × 10 + 50. ROC analyses allowed calculation of the optimal sensitivity and optimal specificity using different cut-off scores of the adjusted scores (T scores).

## Results

### Demographic and neuropsychological variables

**Figure 2:**
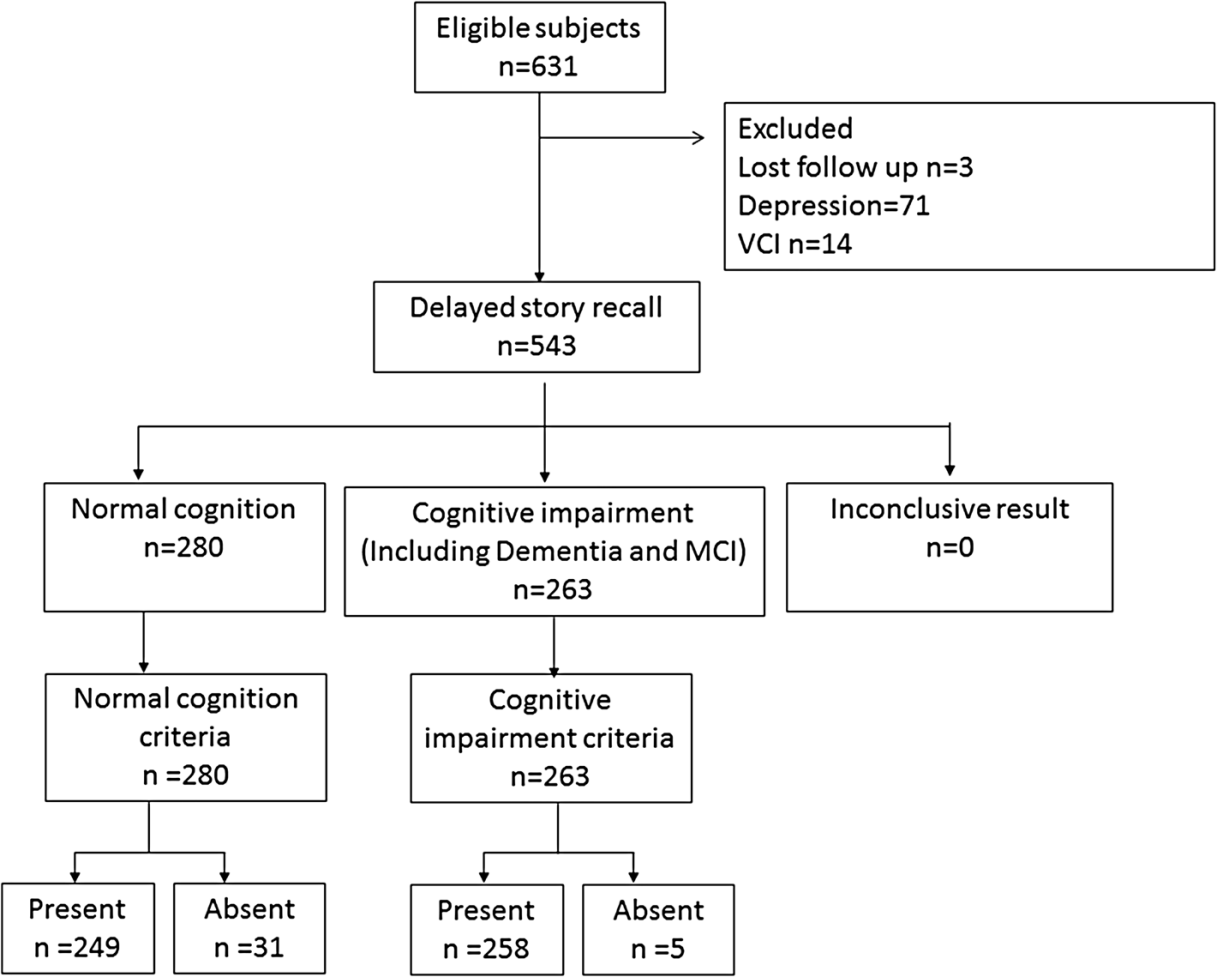
**Standard study flow chart.** Notes: MCI = Mild cognitive impairment; VCI = Vascular cognitive impairment.

A total of 631 subjects were enrolled. Three patients were excluded because they did not complete the neuropsychological assessment, 71 were diagnosed with depression, 14 were considered as having vascular cognitive impairment (VCI), 14 exhibited vascular dementia(VaD), and 50 had a diagnosis of other types of dementia. 249 were classified as NC, 134 as MCI, and 97 as AD. The “All types of dementia group” includes the AD (n = 97), VaD (n = 14), and mixed dementia (n = 50) groups. The study subjects flow chart was shown in Figure 2.

The characteristics of the NC, MCI, AD and all types of dementia groups can be seen in Table 1. There were significant group differences in age and education. The AD and all type of dementia group were significantly older than the MCI group (P = 0.000), and had fewer years of education (P = 0.000). In addition, the MCI group was significantly older than the NC group (P = 0.000) and had fewer years of education (P = 0.000).

**Table 1:** Demographic and neuropsychological characteristics of participant groups

**Group**	**NC n = 249**	**MCI n = 134**	**AD n = 97**	**All type of dementia n = 161**	**X**^**2**^	***P***
Age	66.94 ± 8.87	69.91 ± 8.38**	71.09 ± 9.04**▲▲	70.93(9.15)**	20.052	0.000
Education	12.89 ± 3.30	11.32 ± 3.84**	10.63 ± 4.49**▲▲	10.55(4.39)**	23.622	0.000
MMSE	28.41 ± 1.50	26.98 ± 2.04**	15.69 ± 5.91**▲▲	16.28 (6.06)**△△	255.721	0.000
HAMD	3.78 (3.30)	3.84 (3.12)	2.42 (2.12)	2.78 (2.47)	17.484	0.001
Sex (Male/Female)	158/91	76/58	54/43	81/80	2.597	0.273
Race (Han/Others)	238/11	127/6	92/4	153/7	0.016	0.993
Concomitant diseases						
Hypertension	107	62	30	63	5.727	0.126
Hyperlipidemia	93	33	8	21	46.632	0.000
Diabetes	53	21	14	30	2.828	0.419
Stroke	57	36	13	65	26.287	0.000
History of smoking	45	26	12	39	5.681	0.128
History of drinking	30	19	11	29	3.543	0.315
Family history of dementia	42	27	20	29	1.105	0.776
Story recall test						
ISR	30.03 ± 8.58	13.91 ± 8.79**	4.02 ± 6.06**▲▲	4.49 ± 5.964**▲▲	440.559	0.000
DSR	26.60 ± 8.49	9.40 ± 8.88**	1.60 ± 4.58**▲▲	1.63 ± 4.315**▲▲	460.195	0.000

Notes: Means ± SD was presented except for sex. **P ≤ 0.01 MCI/AD vs NC; ▲▲P ≤ 0.01 MCI vs AD.

NC = normal cognition; MCI = mild cognitive impairment. AD = Alzheimer’s disease. MMSE = Mini-mental State Examination; DSR = Delayed story recall; ISR = Immediate story recall; HAMD = Hamilton depression rating scale.

MMSE, DSR and ISR scores in the AD group and all type of dementia group were significantly lower than in the NC group and MCI group (P = 0.000, P = 0.000, P = 0.000), and those of the MCI group were significantly lower than in the NC group (P = 0.000, P = 0.000, P = 0.000). There was no significant difference between the four groups in terms of sex, race, history of smoking and drinking, and family history of dementia. There was no difference between the four groups regarding concomitant diseases, expect hyperlipidemia and stroke.

Using data from the patients who underwent neuropsychological assessments we entered age and education and HAMD scores into a multiple linear regression analysis with DSR score as the dependent variable. This model overall was statistically significant (F = 12.338, P = 0.000), and can account for 13.3% of the total DSR scores. Examination of the sum of squares for each term in the model, showed that age contributed to the models, and the standardized coefficients were −0.206(P = 0.001). Years of education also had a significant impact on the DSR score (r = 0.746, P = 0.000).

### Normative data as a function of age and education

Given the significant effect of age and education on story recall performance, the present normal control sample’s performance was divided into three age-groups: 50–64 years, 65–74 years and 75–85 years and these are presented in Table 2.

**Table 2:** Normative data for story recall stratified by age and education

**Age 50-64**									
**Education (years)**		**≤9 (n = 23)**		**>9 (n = 54)**			**Total subjects (n = 77)**		
	Mean ± SD	Percentiles		Mean ± SD	Percentiles		Mean ± SD	Percentiles	
		5th	10th		5th	10th		5th	10th
ISR	29.04 ± 9.70	8	20	33.04 ± 8.31	16.0	19.0	31.18 ± 8.89	16.2	20.0
DSR	24.48 ± 8.94	3.75	16.0	30.46 ± 7.99	13.0	18.0	28.10 ± 8.54	13.4	18.0
Percentage story retained (DSR/ISR × 100%)	81.47%			92.79%			90.07%		
**Age 65-74**									
**Education (years)**	**≤9 (n = 22)**			**>9 (n = 47)**			**Total subjects (n = 69)**		
	Mean ± SD	Percentiles		Mean ± SD	Percentiles		Mean ± SD	Percentiles	
		5th	10th		5th	10th		5th	10th
ISR	28.41 ± 10.98	7.35	15.6	31.47 ± 7.33	19.0	21.0	30.16 ± 8.43	17.75	19.5
DSR	23.91 ± 9.14	10.0	10.6	28.43 ± 7.12	13.0	16.0	26.22 ± 8.92	12.0	15.0
Percentage story retained (DSR/ISR × 100%)	87.32%			90.73%			87.37%		
**Age 75-85**									
**Education (years)**	**≤9 (n = 12)**			**>9 (n = 27)**			**Total subjects (n = 39)**		
	Mean ± SD	Percentiles		Mean ± SD	Percentiles		Mean ± SD	Percentiles	
		5th	10th		5th	10th		5th	10th
ISR	25.17 ± 7.58	13.0	13.6	29.19 ± 7.05	13.7	15.8	27.75 ± 7.95	13.5	15.0
DSR	22.58 ± 7.79	11.0	12.2	25.37 ± 8.26	10.5	12.8	24.42 ± 8.90	11.0	13.0
Percentage story retained (DSR/ISR × 100%)	82.59%			85.79%			85.90%		

Note: ISR = Immediate story recall; DSR = Delayed story recall.

### Story recall discrimination of cognitive impairment (MCI and all type of dementia) from NC

**Figure 3:**
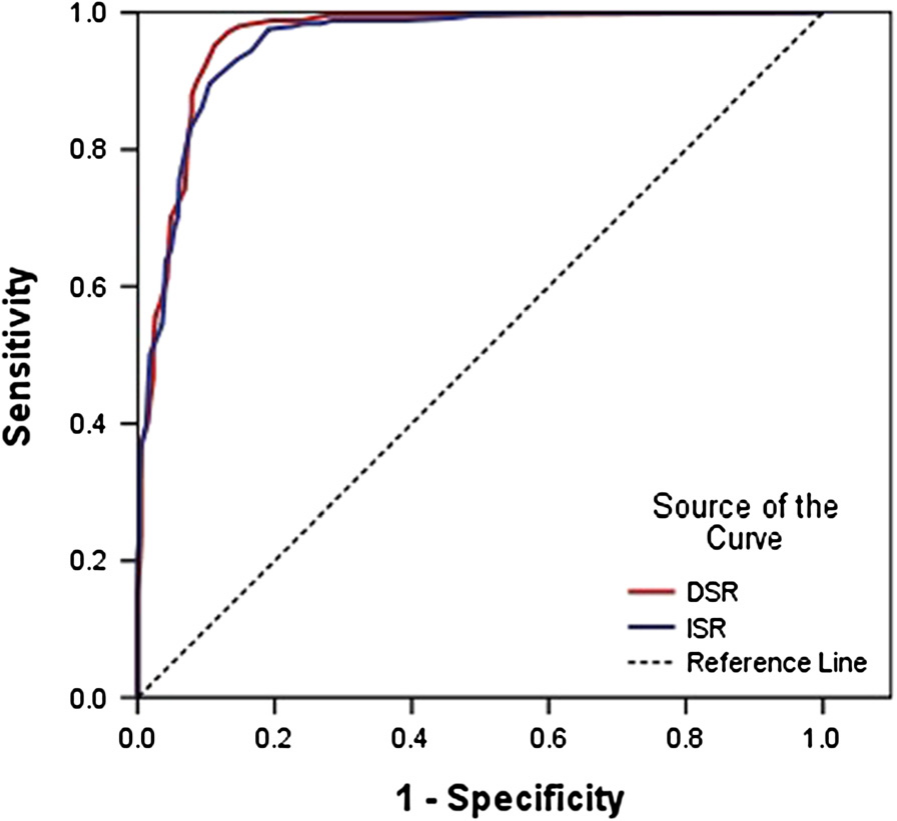
**Receiver operating characteristic curve of story recall in the differentiation of cognitive impairment (mild cognitive impairment and all type of dementia) from normal cognition.** Notes: ISR = Immediate story recall; DSR = Delayed story recall.

We calculated the sensitivity and specificity of ISR and DSR for distinguishing subjects with cognitive impairment (MCI and all types of dementia groups) from the NC group (Figure 3). The area under the curve (AUC) was 0.960 (P = 0.000) (95% confidence intervals(CI):0.946 ~ 0.974) for DSR and 0.954 (95% CI: 0.940 ~ 0.969) for ISR. When the cutoff score was 12.5 for DSR, an optimal balance was obtained between the sensitivity and specificity (95.2% and 88.8%, respectively) in distinguishing cognitive impairment and NC.

### Story recall discrimination of MCI from NC

**Figure 4:**
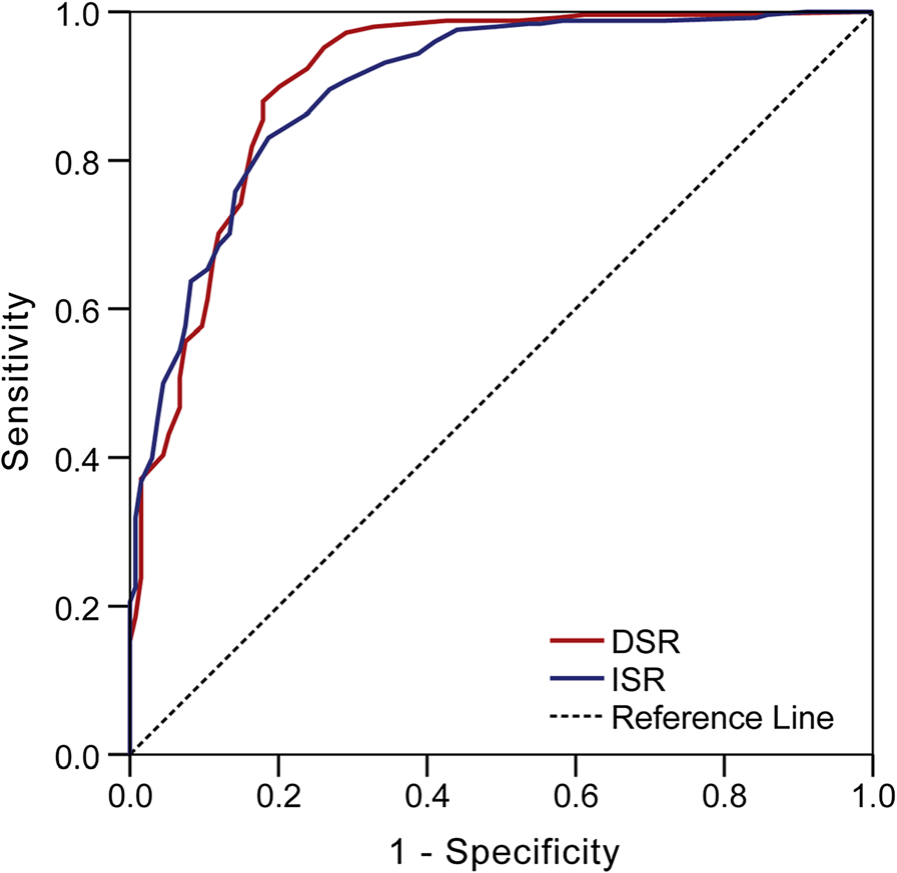
**Receiver operating characteristic curve of story recall in the differentiation of mild cognitive impairment from normal cognition.** Notes: ISR = Immediate story recall; DSR = Delayed story recall.

MCI, especially, aMCI is generally recognized to represent the early-stage of dementia. Hence, we compared subjects with normal cognition with those diagnosed with MCI. The ROC curves were produced by plotting the sensitivity against the 1-specificity for each score on the DSR and ISR for MCI cases versus NC. The area of curve (AUC) of ISR and DSR was 0.898 (95% CI: 0.865 ~ 0.930) and 0.908 (95% CI: 0.875 ~ 0.941) respectively. When the cutoff score was 15.5, the DSR obtained optimal sensitivity and specificity (0.899 and 0.799) for discriminating MCI from NC (Figure 4).

As DSR scores were impacted by age and education, cutoff scores on the DSR were calculated stratified by age and education. The cutoff scores and diagnostic values are shown in Table 3.

**Table 3:** Cutoff scores and diagnostic value of the DSR for discriminating AD and MCI

	**MCI**			**AD**		
	**DSR cutoff**	**Sensitivity**	**Specificity**	**DSR cutoff**	**Sensitivity**	**Specificity**
**Mean**	15.5	0.899	0.799	10.5	0.980	0.938
**Age 50-64**	**15.5**	0.922	0.714	12.5	0.973	0.960
**Education ≤9**	15.5	0.917	0.898	5.50	1.000	0.917
**Education >9**	17.5	0.886	0.714	13.50	.958	1.000
**Age 65-74**	12.5	0.947	0.815	9.5	0.979	0.900
**Education ≤9**	10.5	1.000	0.945	5.0	1.000	1.000
**Education >9**	13.0	0.909	1.000	10.5	0.970	0.882
**Age >75**	10	0.980	0.585	5.0	1.000	0.900
**Education ≤9**	8.5	0.730	0.806	3.5	1.000	0.905
**Education >9**	10.5	1.000	0.904	9.5	0.973	0.974

Note: AD = Alzheimer’s Disease; DSR = Delayed story recall; MCI = Mild cognitive impairment.

### Story recall discrimination of AD or dementia from NC

We compared NC subjects with those diagnosed with AD by a ROC curve. The AUC was 0.986 (0.974 ~ 0.999) for the DSR for detecting AD and 0.988 (95% CI: 0.978 ~ 0.998) for detecting all type of dementia. And 0.988 (95% CI: 0.970-0.998) for ISR for detecting AD and 0.984 (0.972 ~ 0.996) for detecting all type of dementia. A DSR cutoff score of 10.5 yielded an optimal sensitivity and specificity of 0.980 and 0.938 respectively for discriminating AD and NC. The discrimination between NC and AD stratified by age and education was also calculated, as shown in Table 3.

Based on our formula, we calculated the sensitivity and specificity of the adjusted scores (T scores). When the adjusted score was 36.4874, an optimal sensitivity of 0.894 and specificity 0.775 was obtained to discriminate NC from MCI. When the cutoff score was 36.6669, the sensitivity (0.955) and specificity (0.864) was obtained to screening all type of dementia from NC group.

Based on this sample, the prevalence of MCI was 21.2%, and the prevalence of AD was 14.9%. The PPV and NPV was 0.72 and 0.99 respectively for the detection of AD, the PPV and NPV was 0.57 and 0.96 respectively for the detection of MCI. The sensitivity, specificity, PPV and NPV of DSR for different prevalence rates of AD and MCI are shown in Table 4.

**Table 4:** Sensitivity and specificity of DSR in the discrimination of Alzheimer’s disease and mild cognitive impairment and positive predictive values (PPV) and negative predictive values (NPV) at different base rates

							
**Alzheimer’s disease**			**PPV/NPV at different base rate**				
**DSR cutoff**	**Sensitivity**	**Specificity**	**10%**	**20%%**	**30%**	**40%**	**50%**
6.50	0.992	0.907	0.54/1.00	0.73/1.00	0.60/1.00	0.64/0.99	0.68/0.99
7.50	0.988	0.907	0.54/1.00	0.73/1.00	0.82/0.99	0.88/0.99	0.91/0.99
9.00	0.988	0.918	0.57/1.00	0.75/1.00	0.84/0.99	0.89/0.99	0.92/0.99
*10.50	0.980	0.938	0.63/1.00	0.80/1.00	0.87/0.99	0.91/0.98	0.94/0.98
11.50	0.972	0.938	0.63/1.00	0.80/1.00	0.87/0.99	0.91/0.98	0.94/0.97
12.50	0.952	0.959	0.72/1.00	0.85/0.99	0.91/0.98	0.94/0.97	0.96/0.95
14.00	0.923	0.969	0.77’0.99	0.88/0.98	0.93/0.97	0.95/0.95	0.97/0.93
**Mild cognitive impairment**			**PPV/NPV at different base rate**				
**DSR cutoff**	**Sensitivity**	**Specificity**	**10%**	**20%**	**30%**	**40%**	**50%**
12.50	0.952	0.739	0.29/0.99	0.48/0.98	0.60/0.97	0.71/0.96	0.78/0.94
14.00	0.923	0.761	0.30/0.99	0.49/0.98	0.62/0.96	0.72/0.94	0.79/0.91
*15.50	0.899	0.799	0.33/0.99	0.53/0.97	0.66/0.95	0.75/0.92	0.82/0.89
16.50	0.879	0.821	0.35/0.98	0.55/0.96	0.68/0.94	0.77/0.91	0.83/0.87
17.50	0.855	0.821	0.35/0.98	0.54/0.96	0.67/0.93	0.76/0.89	0.83/0.85
18.50	0.819	0.836	0.36/0.98	0.56/0.95	0.68/0.92	0.77/0.87	0.83/0.82
19.50	0.782	0.843	0.36/0.97	0.55/0.94	068/0.90	0.77/0.85	0.83/0.79

Note: *indicates the cutoff with optimal sensitivity and specificity.

### Partial correlation between MMSE and DSR

Given that age and education impact on DSR, we calculated the correlation between MMSE and DSR, controlling for age and education. DSR scores were significantly correlated with MMSE scores (r = 0.575, P = 0.000).

### Test -retest reliability of DSR

56 MCI patients were re-assessed 3 months from baseline, using the same neuropsychological tests. Re-test reliability was calculated by analyzing the correlation between baseline and 3-month scores. The DSR showed high retest reliability (r = 0.887, P = 0.011). The higher the correlation between two evaluations the greater the reliability of the test in the diagnosis of MCI.

## Discussion

To date, there has been a lack of validation studies of story recall measures conducted in a Chinese population. This study provides evidence that story recall has good sensitivity and specificity in discriminating MCI from normal cognition and from AD. This finding provides further evidence that episodic memory declines at an early stage of AD.

The only difference between the original English and Chinese version of the story recall test was a change of name to “Shuzhen Wang”(王淑珍), which is one of the most popular names in Chinese.

Multiple linear regression analysis was used in this study, and the results showed that age and education contributed to the DSR total score. Age showed a negative correlation whereas education level showed a positive correlation with DSR scores. These findings are consistent with those of the original version of the AMIPB story recall test: it was noted that scores on the DSR should be adjusted for age and education.

In the present study, we calculated the sensitivity and specificity of DSR to discriminate MCI or AD from NC. The DSR had a high sensitivity (0.899) and specificity (0.799) in the detection of MCI from NC when the cut-off score was 15.5, and when the cutoff score was 10.5, the DSR obtained optimal sensitivity (0.980) and specificity (0.938) in the discrimination AD from NC.

The MMSE has, for many years, been widely used to assess global cognition in clinical settings. A meta-analysis showed that the MMSE had a poor sensitivity of 85.1%, specificity of 85.5%, PPV of 34.5% and NPV of 98.5% in the distinction between AD and NC. But, it had very limited value in making a diagnosis of MCI against healthy controls with modest rule-out accuracy. It had similarly limited ability to distinguish cases of AD from MCI [28]. Hence, the DSR may be more suitable in the detection of MCI or AD compared to the MMSE.

We have provided age and education adjusted normative data for the DSR in this sample. The original version of the test also provided normative data, for an 18 to 75 year old age range. The normative data of the English version were stratified into four age groups: 18–30 years old, 31–45 years old, 46-60 years old, and 61–75 years old. The mean score was 34.1 ± 10.9 in the 46–60 year old group, and 30.7 ± 11.1 in the 61–75 year old group. In this Chinese sample, the total subject cohort was divided into a 50–64 year old group, 65–74 year old group and 75–85 year old group, and each of those groups was further divided according to education. In this Chinese sample, the normative data are 28.10 ± 8.54 in the 50–64 year old group, 26.22 ± 8.92 in the 65–74 year old group, and 24.42 ± 8.90 in the 75–85 year old group. Owing to the different age banding, the normative data are not directly comparable for the English and Chinese versions. Nevertheless, these normative data presented for a sample of the Chinese population, stratified according to age and education ought to be valuable for the memory assessment of clinical conditions including mild cognitive impairment and AD.

According to the MCI criteria, as defined by Petersen, MCI was classified as (1) amnestic MCI(aMCI), which is said to progress preferentially to AD; (2) MCI characterized by slight impairment in multiple cognitive domains (‘multiple‒domain slightly impaired’), which may progress to AD, to VaD, or may even represent a cognitive ageing process that qualifies as normal; and (3) MCI corresponding to an isolated impairment in a single cognitive domain other than memory (‘single‒domain non‒memory MCI’), which may progress to non‒Alzheimer‒type dementia [29,30]. The diagnosis of a MCI was based on memory tests 1.5 standard deviation (SD) below normative values. However, the Petersen criteria did not supply normative values, so that the diagnosis of a MCI remains difficult in clinics. This study supplied normative data for a Chinese population. Using the Petersen MCI criteria formula (cutoff score = norm-1.5SD), the DSR cutoff score would be 15.3 points for the 50–64 year old group, 12.84 points for the 65–74 year old group, 11.0 points for the 75–85 year old group. These cutoff scores was consensus to those calculated by the ROC curve. In this sample, the cutoff socre was 15.5 for 50–64 year old group, and 12.5 for the 65–74 year old group, and 10 for the 75–85 group in to detecting MCI from NC.

One of the important issues is the predictive value of the DSR. The DSR showed a satisfactory PPV and NPV in this sample. The PPV and NPV were calculated with a cutoff score of 15.5 points for distinguishing MCI from NC. Because the PPV and NPV can be influenced by the base rate of the disease, they were calculated based on literature reports that the prevalence of MCI varies greatly from one study to another, ranging from 3% to around 17% of elderly people (>65 years) [31]. Based on a prevalence of 3%, the PPV and NPV were 12.1% and 99.6% respectively for DSR in detecting MCI, 47.8% and 97.5% for detecting MCI based on a prevalence of 17%, and 82% and 85% based on a prevalence of 50%. The lower the base rate the lower is the PPV. The NPVs are very high, suggesting that we can reassure with confidence those persons who have negative results on these assessments, and they may avoid further neuropsychological evaluations. The calculation of PPV and NPV indicates that the DSR is most valuable in the assessment of people at high risk of cognitive impairment.

In this study, multiple regression showed that age and education were related to the DSR score, so we attempted to develop a model that yielded age and education adjusted norms. Using this model we converted the raw score to adjusted score, and a ROC curve was calculated using the adjusted score. This model effectively adjusts for the effects of age and education. However, it may be difficult to apply it in clinical screening, since it would be inconvenient to calculate adjusted scores for every subject.

Finally, it must be pointed out that the diagnosis of dementia and MCI is a clinical one. It must be based on the clinical interview, neurological examination, laboratory results and imaging, as well as neuropsychological test data. The DSR can only be used as a screening tool, not a diagnostic tool.

There are several limitations in this study, including the relatively small sample size of patients, and a short period of follow-up. Moreover, all subjects were enrolled from a memory clinic, and all, including those who proved to be cognitively normal, had subjective cognitive complaint. Hence, the subjects identified as normal control subjects may not be representative of the normal healthy population, subtle abnormalities may have gone undetected, and resulted in a slight reduction in the obtained normative values. Sensitivity may increase if normative data are obtained from healthy controls from within the general population.

Hence, further studies should be conducted on a larger scale, with longer-term follow-up, and using population-based healthy controls, in order to evaluate the predictive value of the DSR.

## Conclusion

The DSR can be used as a screening tool to detect MCI and AD with high sensitivity and specificity. The DSR correlates with MMSE, and shows high test-retest reliability. The current data show that it is a sensitive screening tool for detecting MCI and AD in the Chinese population. The influence of age and education should be considered in the use of this tool. Additionally, due to the low positive predictive value in people with a low incidence of cognitive impairment, it could be used to identify people with a high risk of cognitive impairment.

## Abbreviations

AD: Alzheimer’s disease; ADL: Ability of daily living; aMCI: Amnestic mild cognitive impairment; CDR: Clinical dementia rating scale; DSR: Delayed story recall; MCI: Mild cognitive impairment; MMSE: Mini-mental State Examination; NC: Normal cognition; PPV: Positive predictive value; NPV: Negative predictive value; ROC: Receiver operating characteristic analysis curve.

## Competing interests

The authors declare that they have no competing interests.

## Authors’ contributions

JT was the study lead, responsible for the design of the study protocol, supervising the data analysis and interpretation of the data, and writing and finalizing the manuscript. JS and MW were the principal investigators for this study, and assisted with designing, drafting, and revising the manuscript, JS reviewed the study, revised the manuscript and supplied advice for the paper. XZ, JN, TLi, WJ, CM, YT, JL, TLiu, PW enrolled patients and conducted the neuropsychological assessments. Y Wang co-designed and conceived the study, was a reviewer of the design of the study protocol. All authors read and approved the final manuscript.

## Pre-publication history

The pre-publication history for this paper can be accessed here:

http://www.biomedcentral.com/1471-244X/14/71/prepub
